# Determination of the critical values of flow parameters characteristic of the alignment of cylindrical nano-objects in suspensions

**DOI:** 10.1016/j.mex.2017.09.002

**Published:** 2017-09-21

**Authors:** Alexander A. Loshkarev, Maria F. Vlasova, Natalya I. Sapronova, Yuri M. Tokunov, Ivan A. Volkov, Victor V. Ivanov, Thomas Maeder

**Affiliations:** a Moscow Institute of Physics and Technology (State University), Institutskii per. 9, Dolgoprudny, Moscow Region 141701, Russia; b École Polytechnique Fédérale de Lausanne, BM 3108 (Bâtiment BM), Station 17, CH-1015, Lausanne, Switzerland

**Keywords:** Ultrasound attenuation, Longitudinal viscosity, Suspension, Cylindrical nano-objects, Alignment, Critical parameters

## Abstract

A method for determining the critical values of the flow speed and the flow constriction degree characteristic of the alignment of cylindrical nano-objects in a flowing suspension is proposed. Previously, the alignment process of cylindrical nano-objects in suspensions was investigated by using birefringence of the polarized light and the small-angle X-ray scattering. While both methods are suitable for measuring the alignment degree of cylindrical nano-objects in suspensions diluted down to low concentrations, they are restricted for the application to undiluted concentrated suspensions because of non-transparency and multiple scattering of X-rays. In addition, the use of the second method requires an expensive synchrotron equipment. We present a simple and faster method based on the direct ultrasound attenuation measurements of longitudinal viscosity of a suspension containing cylindrical nano-objects, which decreases monotonically, approaching its asymptotic value with increase in the flow speed and the flow constriction degree. The principle and advantages of the proposed method are as follows:

• The cylindrical nano-objects align along an accelerated flow at overcritical values of the flow speed and the constriction degree.
• The critical values correspond to the state of a suspension possessing viscosity close to the asymptotic value.
• The method is applicable to undiluted concentrated suspensions, including opaque ones.

## Graphical abstract

**Figure:**
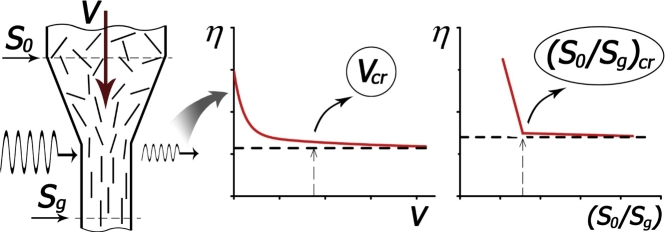

## Method

The given method is a realization of the well-known methodology of ultrasound attenuation spectroscopy [1] for determining the critical parameters of the flow, the flow speed *V* and the flow constriction degree *Z* = *S*_0_/*S*_*g*_, above which the degree of orientation of cylindrical nano-objects in a suspension along the flow is approaching its maximum value (parallel alignment). As an experimental test of forming the oriented state of cylindrical nano-objects in the suspension flow, the direct ultrasound attenuation measurements of longitudinal viscosity *η* were used [2].

Two other techniques allowing to control the orientation of cylindrical nano-objects in suspensions are reported in papers [3], [4]. The first one is based on the effect of rotation of the plane of polarization of linearly polarized light as it travels through the suspension containing oriented cylindrical nano-objects: the higher is the degree of alignment of nano-objects, the higher is the light intensity. This method can be applied to the transparent suspensions only with the concentration of colloidal objects below 0.3–1.0 wt.% depending on the material. The second technique is based on small-angle X-ray scattering (SAXS). Similarly to the first technique, the second one can be applied to quite dilute suspensions; also because of the need to use synchrotron radiation that enables to achieve higher signal-to-noise ratio, it becomes time-consuming and expensive for users. The proposed method offers two advantages over these common techniques: i) simplicity of application due to the availability and mobility of acoustic instruments; ii) applicability to concentrated suspensions (0.3–70.0 wt.%), including opaque ones.

The theoretical background of the experiment consists in the decrease in the longitudinal viscosity of the suspension containing cylindrical nano-objects in the course of their alignment along the direction of a flow as a result of reduced interaction between the parallel layers of a suspension.

**Fig. 1:**
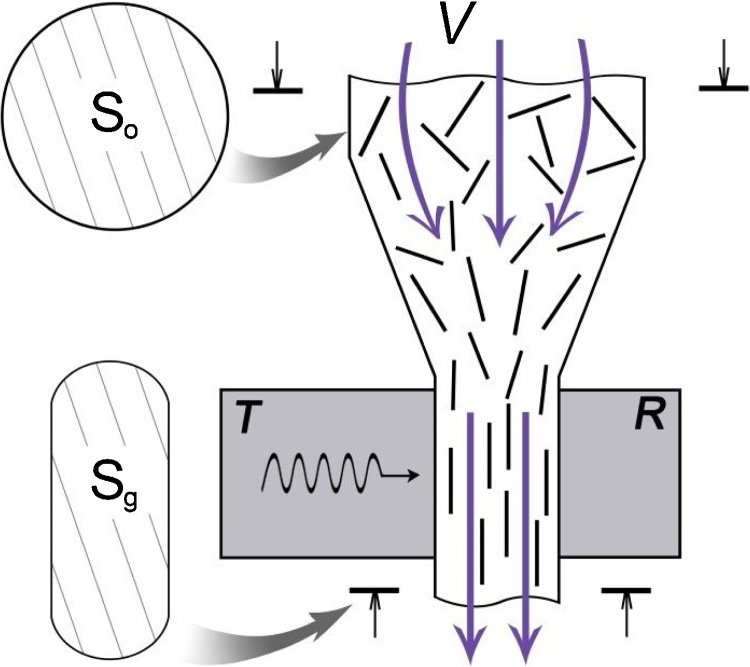
Scheme of the measurement cell. The suspension flows from the round-shaped cross-section with the area *S_0_* to the flattened cross-section with the area *S_g_* located in the measurement zone of an acoustic sensor. The direction of the flow is shown by arrows. While entering the constricted channel, the cylindrical nano-objects are beginning to align along the accelerated flow. The ultrasonic wave is propagating from the transmitter T to the receiver R through a suspension in the direction perpendicular to a flow.

In the flow-type measurement cell of the acoustic spectrometer DT-500 (Dispersion Technology, USA), the measurements of the acoustic attenuation spectra and the longitudinal viscosity of the flowing suspension were carried out at different values of the flow speed and the flow constriction degree at the filler neck (Fig. 1). The flexible measurement cell made of silicon rubber tube has a crucified form with four branch pipes, two of which serve for connecting the closed-loop circular channel with the inner diameter *D*. Two other branch pipes are intended for plugging the transmitter ***T*** and the receiver ***R*** of the acoustic sensor so that the positions of their end faces coincide with the inner wall of the circular channel. The suspension under study is poured into the channel and put into motion by the peristaltic pump with the adjustable speed of the flow. The ultrasonic wave is propagating from the transmitter to the receiver through a suspension containing cylindrical nano-objects aligned preferably along the direction of the flow accelerated in the constricted channel of the measurement cell.

Due to the flexibility of the silicon rubber tube, the transmitter can move relative to the receiver during the measurements. As a result, the cross-section of the channel in the measurement zone represents a flattened circle with the width *b* corresponding to the gap between the transmitter and the receiver. The cross-section of the rest part of the channel has a circular shape. The thermostat being a part of the setup makes it possible to maintain the temperature of the suspension in the range from 29 to 31 °С.

In the constricted area located between the main channel with the cross-section *S*_0_ = *πD*^2^/4 and the measurement zone with the cross-section *S*_*g*_, which is much less than *S*_0_, the accelerated flow of the suspension under study is formed. The longitudinal flow velocity gradient determines the average angular speed $\dot{\phi }$ of long cylindrical nano-objects rotating its axes towards the flow direction [5], [6]:

(1) $$\dot{\phi }=-\frac{3}{4}\frac{\partial V}{\partial x}\left(\frac{A^{2}-1}{A^{2}+1}\right)\sin\left(2\phi \right)$$

Having passed through the area of accelerated flow, the colloidal nano-objects acquire the resulting degree of orientation determined (without taking into account the influence of the Brownian motion and the weak influence of the aspect ratio *A*) by the flow constriction degree *Z*. The last one is defined as follows:

(2) $$Z=\frac{S_{0}}{S_{g}}=\frac{D}{2b}{\left(1-\frac{b}{2D}\right)}^{-1}$$

The Eq. (1) allows one to estimate the values of the flow constriction degree corresponding to the perfect alignment of nano-objects: *Z* ≥ 2, 5. However, this condition is not sufficient because of the influence of the decay mechanism associated with the Brownian rotation of nano-objects. The effect of the rotational diffusion can be ignored provided that the characteristic time of the alignment of colloidal objects and the time required for them to pass through the measurement gap are less than that of the significant diffuse reversal of nano-objects [3], [7].

Therefore, the first step of the proposed method consists in the measurement of the dependence of the longitudinal viscosity on the flow speed of a colloid η(V) at Z values greater than 2.5. The approaching of the viscosity to the limiting value *η*_∞_ should imply the validity of the assumption of neglect of the Brownian rotation. The flow speed value at which the difference between the viscosity and its limiting value is equal to 5% can be considered as a critical speed value *V_cr_*.

The second step of the method is to carry out measurements in order to find the relationship between the longitudinal viscosity and the flow constriction degree at a fixed value of the flow speed close to its critical value *V* > *V_cr_*. The η(Z) dependence should represent a monotonically decreasing function asymptotically approaching the limiting value *η*_∞_. The flow constriction degree value at which the difference between the viscosity and its limiting value is equal to 5% can be considered as a critical flow constriction degree value *Z*_*cr*_.

Thus, it is possible to determine the critical values of flow parameters of a colloid, *V_cr_* and *Z*_*cr*_, beyond which the cylindrical nano-objects contained therein align perfectly along the flow direction.

### Method validation

As an example, we describe in details the experimental procedure of determining critical values of parameters of the flow of the aqueous colloid of carbon nanofibers Palizh-100 provided by the company Noviy Dom, LLC (Izhevsk, Russia). To remove large agglomerates of nano-fibers from the sample, it has been subjected to intense sonication with the acoustic power of 100 W for 40 min followed by the rapid sedimentation procedure in the centrifugal field of 5000 ÷ 7000 g for 10 ÷ 30 min by using the high-speed centrifuge. The content of carbon nano-objects in the sample under study was about 3.8 wt.%; its sedimentation stability was sufficient for performing acoustic measurements. The average size parameters (diameter, length_,_ and the aspect ratio) of carbon nanofibers were estimated at 9.5 nm, 860 nm and 90, respectively, as found from the transmission electron microscopy images obtained with JEOL JEM-2100.

**Fig. 2:**
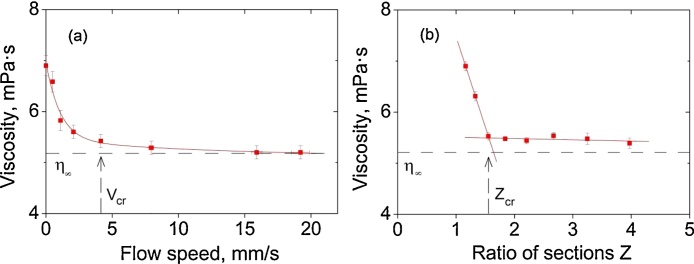
Dependences of longitudinal viscosity of the aqueous suspension of carbon nanofibers: (a) – on the flow speed at a flow constriction degree of 3.25; (b) – on the flow constriction degree at a flow speed of 4.5 mm/s. The critical values of the flow speed and the flow constriction degree are estimated at 4.5 mm/s and 1.6, respectively.

In the first series of experiments performed at a fixed value of the flow constriction degree (*Z* > *Z_cr_*) we observed the asymptotic decrease in the longitudinal viscosity with the increase in the flow speed in the round section of the channel (Fig. 2a). The estimated limiting value is *η*_∞_ = 5.2*mPa* * *s*. It can be seen from Fig. 2a that at flow speeds above 4 mm/s the η(V) dependence has a slight slope with the trend of saturation. Such a behavior of the viscosity indicates that the cylindrical colloidal objects tend to attain the ultimate degree of orientation (parallel to the direction of the flow of a suspension).

In the second series of experiments at a fixed value of the flow speed in the round section (*V* > *V_cr_*), we performed measurements of the longitudinal viscosity as a function of *Z* in the range from 1.16 to 16.25. Fig. 2b presents the η(Z) dependence. One can see from this figure that the longitudinal viscosity has the asymptotic behavior in the range Z >* Z_cr_* that is also indicative of the perfect orientation of carbon nanofibers under such conditions. It is worth noting that the Reynolds number during the experiments did not exceed 80 that is far below the threshold of the turbulence, thus providing a necessary laminar regime for the flow of suspensions studied.

The results of two series of experiments provide support for the statement that the state of a colloid with the ultimate degree of orientation of nano-objects is attained when the flow speed and the flow constriction degree exceed its critical values.
